# Identification and characterization of three monoclonal antibodies targeting the SFTSV glycoprotein and displaying a broad spectrum recognition of SFTSV-related viruses

**DOI:** 10.1371/journal.pntd.0012216

**Published:** 2024-06-07

**Authors:** Xiaoli Wu, Abulimiti Moming, Yanfang Zhang, Zhiying Wang, Tao Zhang, Liyan Fu, Jin Qian, Jun Ni, Sijing Hu, Shuang Tang, Xin Zheng, Hualin Wang, Shu Shen, Fei Deng

**Affiliations:** 1 Key Laboratory of Virology and Biosafety and National Virus Resource Center, Wuhan Institute of Virology, Chinese Academy of Sciences, Wuhan, Hubei, China; 2 Brain Science and Advanced Technology Institute, Wuhan University of Science and Technology, Wuhan, China; 3 University of Chinese Academy of Sciences, Beijing, China; 4 Department of Infectious Diseases, Union Hospital, Tongji Medical College, Huazhong University of Science and Technology, Wuhan, China; University of Geneva Hospitals, SWITZERLAND

## Abstract

Severe fever with thrombocytopenia syndrome virus (SFTSV) is a novel tick-borne viral pathogen that causes severe fever with thrombocytopenia syndrome (SFTS). The disease was initially reported in central and eastern China, then later in Japan and South Korea, with a mortality rate of 13–30%. Currently, no vaccines or effective therapeutics are available for SFTS treatment. In this study, three monoclonal antibodies (mAbs) targeting the SFTSV envelope glycoprotein Gn were obtained using the hybridoma technique. Two mAbs recognized linear epitopes and did not neutralize SFTSV, while the mAb 40C10 can effectively neutralized SFTSV of different genotypes and also the SFTSV-related Guertu virus (GTV) and Heartland virus (HRTV) by targeting a spatial epitope of Gn. Additionally, the mAb 40C10 showed therapeutic effect in mice infected with different genotypes of SFTSV strains against death by preventing the development of lesions and by promoting virus clearance in tissues. The therapeutic effect could still be observed in mice infected with SFTSV which were administered with mAb 40C10 after infection even up to 4 days. These findings enhance our understanding of SFTSV immunogenicity and provide valuable information for designing detection methods and strategies targeting SFTSV antigens. The neutralizing mAb 40C10 possesses the potential to be further developed as a therapeutic monoclonal antibody against SFTSV and SFTSV-related viruses.

## Author summary

Severe Fever with Thrombocytopenia Syndrome Virus (SFTSV) is a tick-borne pathogen that causes severe illness known as SFTS. This disease has become a major health concern, particularly in China, Japan and South Korea, with a high mortality rate. Regrettably, there are currently no vaccines or effective treatments available for this disease. To address this critical gap, we developed three monoclonal antibodies (mAbs) targeting a specific region of the SFTSV glycoprotein. Among these mAbs, one, named 40C10, exhibited remarkable neutralizing activity against SFTSV as well as related viruses such as HRTV and GTV. It provided complete protection against disease and death in mice even when administered days after viral exposure. These findings are promising and suggest that mAb 40C10 has potential as a therapeutic option against SFTSV. This work is a significant step towards meeting the urgent need for effective treatments for SFTS.

## Introduction

Severe Fever with Thrombocytopenia Syndrome (SFTS) is an emerging tick-borne disease. Its main symptoms include fever, thrombocytopenia, dizziness, nausea, and multiple organ failure, with a fatality rate of up to 30% [1,2]. Given the lack of specific drugs and vaccines, SFTS was included in the list of priority diseases for urgent research by the World Health Organization (WHO) in 2017. The SFTS virus (SFTSV) belongs to the order *Bunyavirales*, family *Phenuiviridae*, and genus *Bandavirus* (https://talk.ictvonline.org/taxonomy/), and was identified as the causative pathogen of the disease and was first isolated from the serum samples of a patient with SFTS in China in 2009 [3]. SFTSV was mainly prevalent in central and eastern China, Japan and South Korea, where reported human cases confirmed with SFTSV infection [4,5]. Serological evidence also suggested SFTSV prevalence and occurrence of human exposure in Pakistan [6] and Vietnam [7], indicating that the virus may be widely distributed in East Asia. *Haemaphysalis longicornis* is considered the primary vector of SFTSV due to reports with a history of tick bites or outdoor activities and ticks collected from the vicinity of patient’s residence tested positive for the virus [8]. SFTSV can also be transmitted from human-to-human, particularly within families where caregivers often lack protective gear [9,10]. Additionally, there have been documented cases of SFTS in humans who have been in contact with SFTSV-infected cats [9]. After the identification of SFTSV, a viral pathogen closely related to SFTSV was identified in the United States from febrile patients with clinical symptoms similar to SFTS and was named Heartland virus (HRTV) [10]. In 2016, Guertu virus (GTV) was identified in *Dermacentor nuttalli* ticks collected in Xinjiang, China, and is phylogenetically associated with both SFTSV and HRTV. The identification of neutralization activity in some healthy humans against GTV suggested that prior exposure to GTV had occurred, thus posing a potential threat of infection to humans [11].

SFTSV is a three-segment negative-sense RNA virus whose genome contains large (L), medium (M), and small (S) segments [2]. The L segment encodes the RNA-dependent RNA polymerase (RdRp), the M segment for an envelpoe glycoprotein (GP) cleaved into the N-terminus fragment (Gn) and the C-terminus fragment (Gc), which are targets for neutralizing antibodies [12,13] and crucial for receptor binding [14], and the S segment for nonstructural proteins (NSs) and nucleoproteins (NPs). Based on sequence comparison of the three RNA segments, SFTSV strains can be divided into the Chinese (C, C1-C5) lineage, which mainly contains strains from China, and the Japanese (J, J1-J3) lineage, containing most of the strains from Japan and South Korea, suggesting that SFTSV phylogeny is associated with geographical distribution [4]. Previous studies have reported the emergence of new genotypes, such as C6 and J4, and chimeric strains containing three segments of two or three different genotypes [15,16]. The increasing genetic diversity and complexity of the SFTSV genotypes may pose challenges in the design of SFTSV detection methods and antiviral strategies.

Monoclonal antibodies (mAbs) recognize viruses and can be used in the development of detection kits and in research on macromolecular drugs. Neutralizing mAbs can neutralize viruses and reduce the severity of infection, and thus could be developed as candidates in clinical treatments [17,18]. Three neutralizing mAbs (Ab10, MAb4-5 and SNB02) that recognize different epitopes locating in SFTSV Gn have been reported [19,20], which can neutralize SFTSV *in vitro* and *in vivo* [21]. Developing specific neutralizing mAb drugs is crucial for blocking SFTSV spread and understanding infection mechanisms. Investigating broad-spectrum effects of mAbs against diverse SFTSV strains is equally important.

In this study, three mouse-derived mAbs were developed and characterized using hybridoma technology, targeting SFTSV Gn. Two out of the three mAbs recognized specific epitopes on the SFTSV Gn, while the third mAb exhibited a broader reactivity to the spatial structure of SFTSV Gn, neutralizing different genotypes of SFTSV strains. The protective efficacy of the mAbs was assessed by monitoring survival rates, changes in body weight and tissue lesions in SFTSV-infected mice. Additionally, the cross-neutralizing activity of mAbs against SFTSV-related viruses were investigated. This study provides insights into the generation of SFTSV-specific mAbs and identifies a neutralizing mAb with broad-spectrum activity against SFTSV and related viruses. The potential applications of these SFTSV-specific mAbs are also discussed.

## Material and methods

### Ethical statement

Animal experiments were approved by the ethics committee of Wuhan Institute of Virology, Chinese Academy of Sciences under the approval number WIVA33202006. All cell and animal experiments were performed in Biological safety third-level laboratory (BSL-3) and Animal Biological safety third-level laboratory (ABSL-3). The procedure was approved by the Ethics Committee of Union Hospital of Huazhong University of Science and Technology in Wuhan (approval number: 2018S292).

### Cells, viruses, antibodies, and serum of SFTS patients

Vero (ATCC number: CCL-81) and SP2/0 Myeloma cell lines (ATCC number: PTA-9396) were obtained from the American Type Culture Collection (ATCC, Manassas, VA, USA) and cultured in high-glucose Dulbecco’s Modified Eagle’s medium (DMEM; NZK Biotech, Wuhan, China) supplemented with 10% foetal bovine serum (FBS; Gibco, Australia). Spodoptera frugiperda Sf9 cells were cultured in Grace’s insect medium (Gibco, Grand Island, NY, USA), at pH 6.0, supplemented with 10% FBS at 27°C.

The SFTSV strains were preserved at the National Virus Resource Centre (NVRC, Wuhan, China; preservation number: HBZN15-C1 genotype: CSTR:16533.06. IVCAS 6.9027; HBGS13-C2 genotype: CSTR:16533.06. IVCAS 6.6312 [22]; HBMC5-C3 genotype: CSTR16533.06. IVCAS6.6311 [22]; WCH-2011/HN/China/isolate97-C4 genotype: CSTR:16533.06.IVCAS 6.6088 [23]). The GTV strain DXM (preservation number: CSTR:16533.06. IVCAS 6.6106) was preserved at the NVRC [11]. The HRTV strain (strain MO-4, CSTR:16533.06. IVCAS 6.6330) were obtained from the World Reference Centre for Emerging Viruses and Arboviruses (University of Texas Medical Branch) and preserved at the NVRC.

Polyclonal antibodies (pAb) prepared in-house, including anti-SFTSV NP [24] and anti-SFTSV Gn [11] were used to detect viral protein expression in cells by western blotting (WB) or immunofluorescence assays (IFAs). The secondary antibodies for WB or IFAs were consistent with previous literature reports [25,26]. Three IgG antibody-positive serum samples from SFTS patients were preserved at the NVRC by the Department of Infectious Disease at Union Hospital (Wuhan City, Hubei Province).

### Screening of monoclonal antibodies

The SFTSV was inactivated by β-propiolactone (SERVA Electrophoresis GmbH, Heidelberg, Germany) as previously described [26]. Female BALB/c mice (6–8 weeks old, n = 6) were immunised with inactivated 10^8^ TCID_50_ SFTSV (200 μL/mouse) through both the intraperitoneal and subcutaneous routes, three times, two weeks apart. The fusion of hybridoma cells and the screening of antibodies were conducted using the same methods as previously described [26].

### Prokaryotic and eukaryotic expression

A prokaryotic truncated expression polypeptide constructed by Moming et al. was used for the epitope identification of mAbs [27]. The gene fragment of truncated SFTSV Gn (amino acid 20 to 452) was cloned into the pFastBac Dual vector (Invitrogen, Carlsbad, CA, USA) under the control of polyhedrin promoter (P_PH_), and then was fused with a strep tag at the C-terminus. The cassette of P_PH_-SFTSV Gn_20-452_-strep was further transposed into Autographa canifornica multiple nucleopolyhedrovirus (AcMNPV) [28] using the Bac-to-Bac system (Invitrogen, Carlsbad, CA, USA), according to the manufacturer’s instructions, to generate the recombinant baculovirus vAc-Gn_20-452_. All constructs were verified using PCR and Sanger sequencing.

### IFA, WB assays, and Micro-neutralization test (NTs)

IFA and WB were performed to screen and identify monoclonal antibodies using Vero cells infected with SFTSV, GTV, or HRTV at a multiplicity of infection (MOI) of 1 TCID_50_ unit per cell, viral particles purified from the cell culture supernatants, or Sf9 cells infected with recombinant baculovirus as antigens as previously described [25,26]. Neutralization activity of monoclonal antibodies were further determined by NTs as previously described [11]. The inhibition effect by monoclonal antibody on different strains was analysed by the GraphPad Prism software version 8.0.2 (San Diego, CA, USA).

### Animal experiments

The effect of monoclonal antibody protecting mice from infection of different strains of SFTSV were investigated using IFNAR^-/-^ C57BL/6 mice. Briefly, the female mice (6-8-week-old, n = 6/group) were intraperitoneally injected with SFTSV (HBGS13, HBMC5 or WCH strain) at a median lethal dose (LD_50_) of 50 per mouse, as the doses quantified by LD_50_ instead of TCID_50_ ensured the lethal outcome in mice after challenge and would benefit comparing analyses of the protection effect from the use of 40C10. Then, the mice were administered with monoclonal antibodies 40C10 or 20C4 (30 μg/g per mice, n = 6/group) four times at 1, 24, 48, and 72 h post infection (p.i.), respectively, and equivalent volume of PBS was used as control. Body weight changes and outcomes were monitored daily for 12 days right after SFTSV infection. Liver, spleen, lungs, and kidneys were collected from mice infected with the HBGS13 strain on days 4 and 12 p.i. (n = 3 at each time point), respectively, and were fixed in 4% paraformaldehyde for 24 h. Hematoxylin-eosin (H&E) staining and immunohistochemical assays were performed to observe the pathological changes caused by SFTSV infection and to blot SFTSV infection in tissues using a pAb against SFTSV NP as previously described [29]. SFTSV RNA loads in tissues and serum samples were determined by qRT-PCR as previously showed [30].

The protective effect of mAbs given to SFTSV infected mice at a later time point was investigated as previously described [20]. Briefly, the female IFNAR^-/-^ C57BL/6 mice were intraperitoneally infected with SFTSV (HBGS13 strain, 20 or 50 LD_50_ per mouse, n = 6/group). The mAb or PBS was given to these mice with the first infection on 1, 2, 3, or 4 days, once a day and lasting for four days. Mice were monitored for 12 days to inspect their body weight changes and outcomes.

## Results

### Three anti-Gn glycoprotein antibodies were identified from immunised BALB/c mice

**Fig 1:**
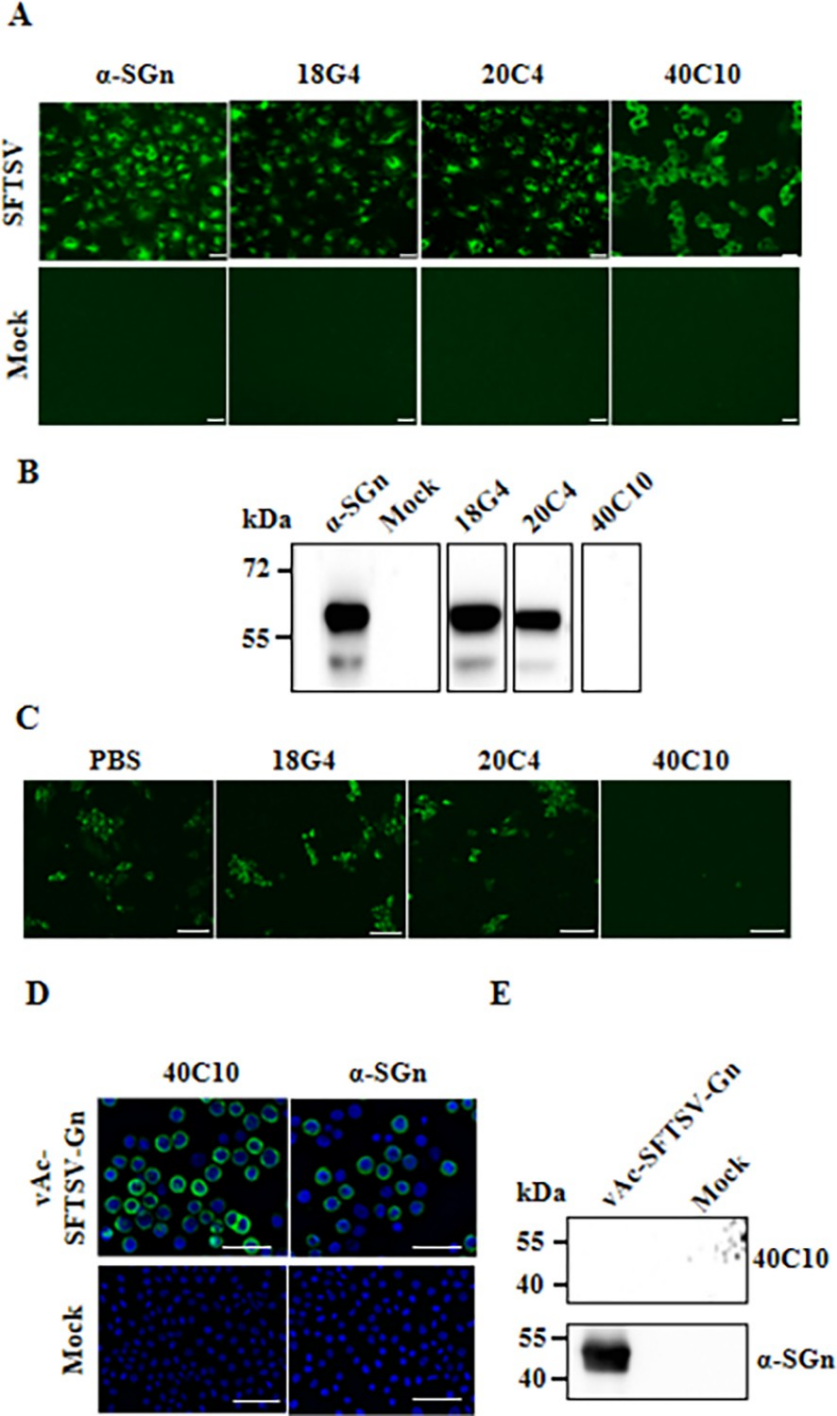
Screening and identification of mAbs. The infected Vero cells with 1 MOI SFTSV (strain: HBMC5-C3 genotype) was detected by IFA (A) and WB (B). The mock cells were uninfected cells. Bar: 50 μm. C. Neutralization evaluation of mAb. Bar: 100 μm. Identification of the 40C10 mAb with recombinant baculovirus (vAc-Gn_20-452_) infected cells using IFA (D) and WB (E). The mock cells were uninfected cells. Bar: 50 μm.

Three mAbs specific to SFTSV antigens were identified. The 18G4 and 20C4 mAbs demonstrated the ability to recognize SFTSV-infected cells in IFA and the SFTSV-Gn protein in WB (Fig 1A and 1B), whereas the 40C10 mAb only exhibited recognition of SFTSV-infected cells and eukaryotic expression of SFTSV-Gn protein through IFA (Fig 1A, 1D and 1E). Furthermore, the results for NTs indicated that the mAb 40C10 had a neutralizing effect on SFTSV, whereas the 18G4 and 20C4 mAbs did not demonstrate neutralizing activity against SFTSV (Fig 1C). Three mAbs were screened by monoclonal antibody screening technique.

### Gn epitopes recognized by the 18G4 and 20C4 mAbs were identified

**Fig 2:**
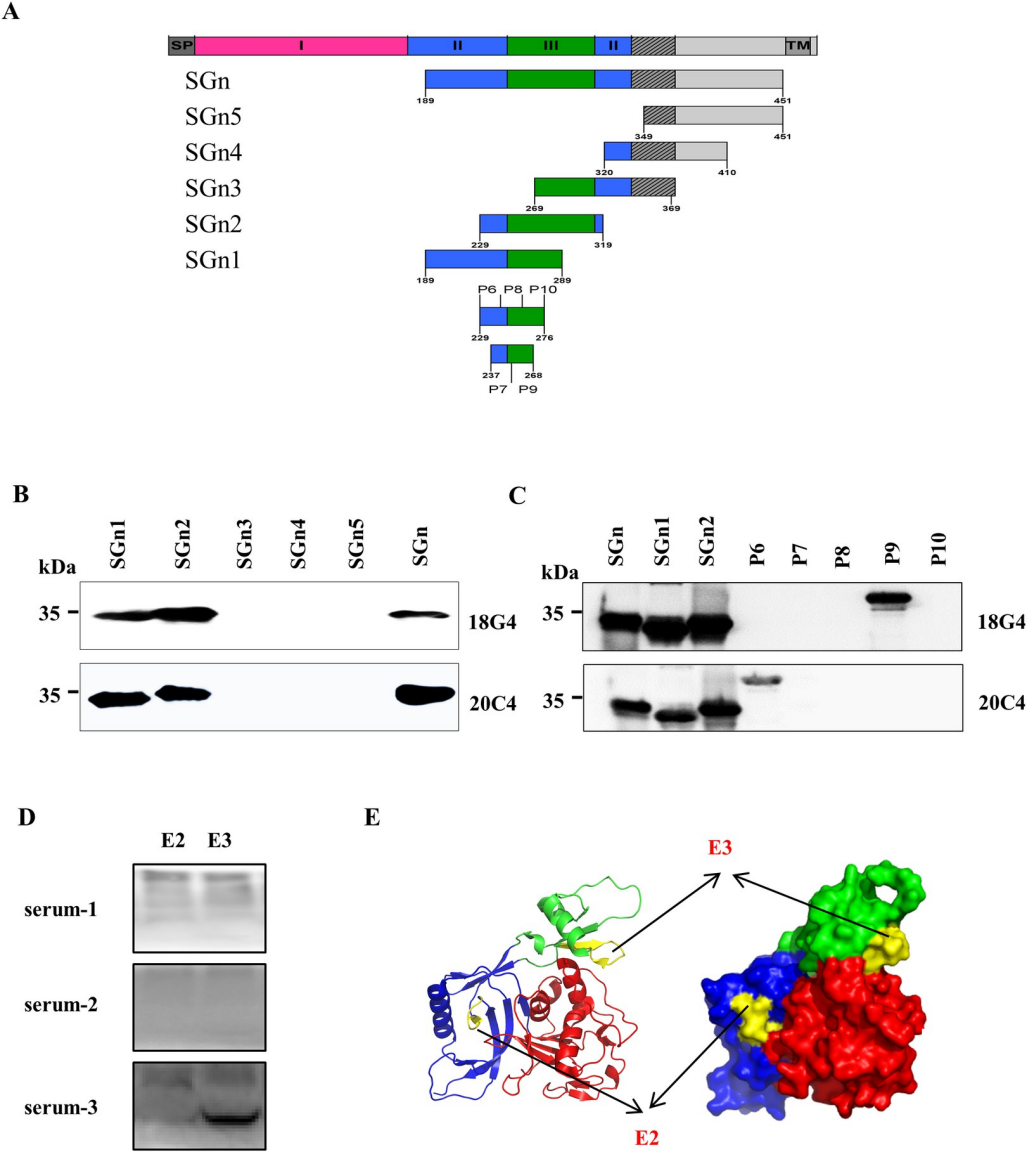
Identification of 18G4 and 20C4 mAb epitopes. A. Antigen truncation diagram. Schematic representation of the full length SFTSV-Gn andtruncated fragments of Gn, including Signal peptide (SP), three domains (I, red; II, blue; and III, green), and Transmembrane region (TM). Schematic representation of truncated fragments of Gn: SGn (189–451 aa), SGn1 (189–289 aa), SGn2 (229–319 aa), SGn3 (269–369 aa), SGn4 (320–410 aa), SGn5 (349–451 aa), P6 (229–244 aa), P7 (237–252 aa), P8 (245–260 aa), P9 (237–252 aa) and P10 (261–276 aa) segments respectively. Identification of 18G4 (B) and 20C4 (C) mAb epitopes by WB. D. E2 and E3 epitopes were recognized by SFTS IgG-positive sera. E. 18G4 (E3) and 20C4 (E2) mAb epitopes were assigned to the 3D SFTSV-Gn structure. The right side of SFTSV-Gn structure was display in different model: surface model (right) and cartoon model (left). Red, domain I; blue, domain II; green, domain III; yellow, epitope E2 and E3.

To determine the specific antigen epitopes recognized by 18G4 and 20C4 antibodies, truncated versions of the Gn were constructed using prokaryotic expression (Fig 2A). WB results revealed that 18G4 and 20C4 mAbs recognized SGn (189-319aa), SGn1 (189-289aa) and SGn2 (229-319aa) (Fig 2B). Further truncation of Gn (189-319aa) revealed that the 18G4 and 20C4 mAbs specifically targeted P9 and P6 of Gn, respectively (Fig 2C). According to Moming [27], the epitopes recognized by 18G4 and 20C4 were E2 (^257^VCYKEGTGPC^266^) and E3 (^233^GHSHKII^239^) in the 3D structure of Gn (Fig 2E). WB analysis demonstrated that serum-3, an IgG antibody-positive serum of SFTS, recognized the E2 and E3 epitopes of SFTSV-Gn (Fig 2D).

### 18G4 and 20C4 mAbs cross-recognized SFTSV and GTV, while 40C10 cross-neutralized SFTSV, GTV, and HRTV

**Fig 3:**
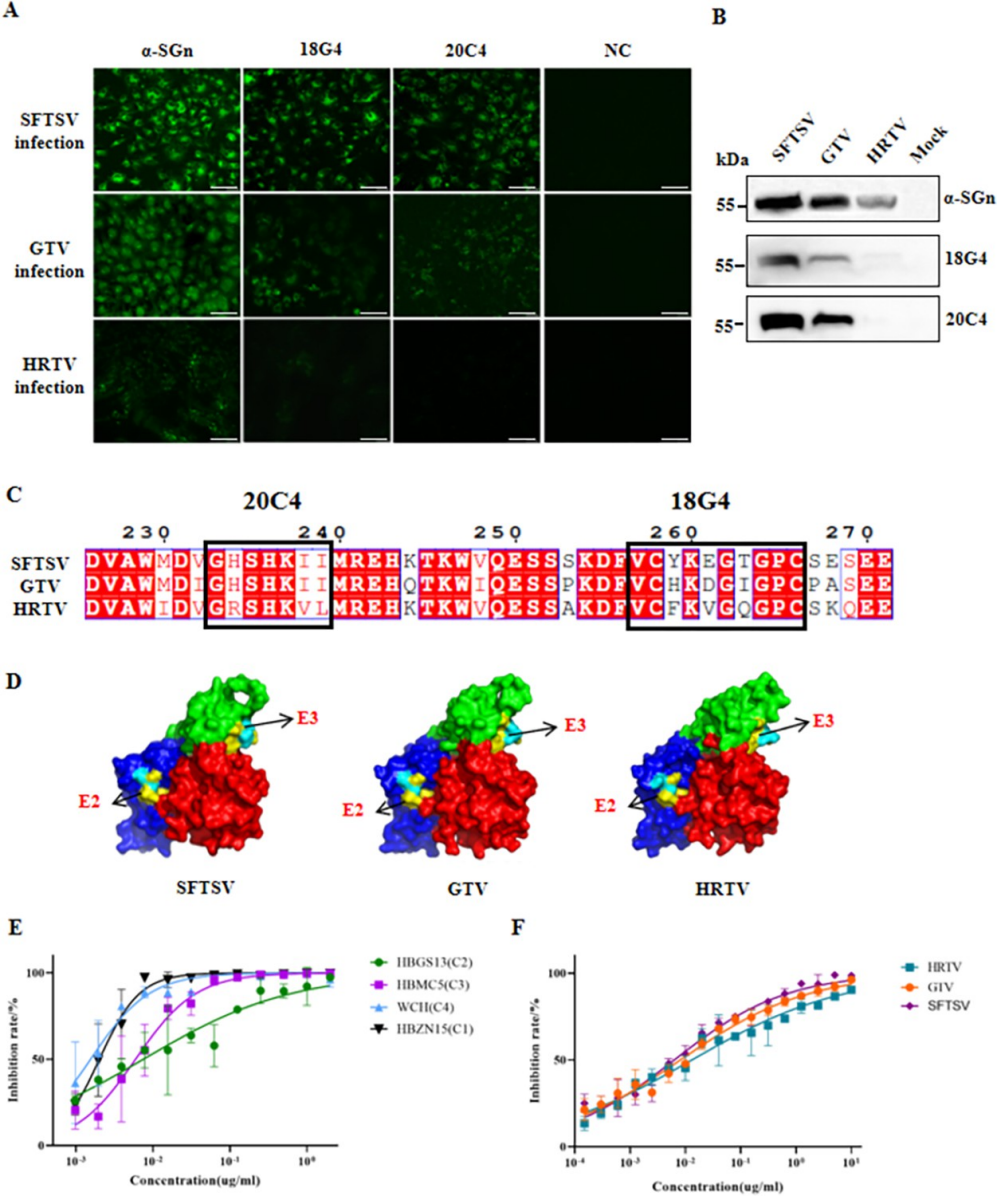
Cross-recognition or cross-neutralization evaluation of mAbs. Evaluation of 18G4 and 20C4 mAb cross-recognition of SFTSV-related viruses using IFA (A) and WB (B). Comparison of epitopes of SFTSV, GTV, and HRTV recognized by 18G4 and 20C4 mAbs by amino acid sequence (C) and 3D structure (D). Red: domain I; blue: domain II, green: domain III; yellow: epitope E2 and E3; light blue: different amino acid on E2 and E3 epitopes of SFTSV, GTV and HRTV. E. The 40C10 mAb neutralized different SFTSV genotypes (HBZN15-C1 genotype, HBGS13-C2 genotype, HBMC5-C3 genotype, WCH-C4 genotype). F. 40C10 neutralized SFTSV-related viruses (GTV or HRTV). All data are presented as mean values ± SD.

We assessed the cross-recognition capability of 18G4 and 20C4 mAbs against Bandaviruses using IFA and WB. The results demonstrated that 18G4 and 20C4 mAbs recognized SFTSV and GTV, but not HRTV. The recognition of GTV by the mAbs was weaker compared to SFTSV (Fig 3A and 3B). The epitopes recognized by the 20C4 mAb in SFTSV and GTV were consistent, while the amino acids at positions 234, 238 and 239 differed between HRTV (R,V and L, respectively) and SFTSV/GTV (H, I and I, respectively) (Fig 2C). The mAb 18G4 recognized antigen epitopes at amino acid sites 259, 261, and 263 in SFTSV\GTV\HRTV, corresponding to Y\H\F, E\D\V, and T\I\Q, respectively (Fig 2C). The distinct amino acids in the E2 and E3 epitopes of SFTSV, GTV, and HRTV were identified in the 3D structure of Gn (Fig 3D).

To assess the neutralizing effect of the 40C10 mAb, various genotypes of SFTSV strains, along with Bandaviruses GTV and HRTV, were employed. The results demonstrated that the mAb 40C10 effectively neutralized different genotypes of SFTSV strains. The neutralizing effects on the SFTSV strains, ranked from strong to weak according to the IC_50_ values from low to high, were as follows: HBZN15 (C1, 2.015 ng/mL) > WCH (C4, 4.360 ng/mL) > HBMC5 (C3, 5.940 ng/mL) > HBGS13 (C2, 7.506 ng/mL). Additionlly, the mAb 40C10 showed neutralizing activity against HRTV and GTV, with IC_50_ values of 13.12 ng/mL and 8.140 ng/mL, respectively (Fig 3E). In this study, the SFTSV strain HBGS13 (C2) was used as the control. The neutralizing abilities for different viruses, ranked from strongest to weakest, were as follows: SFTSV > GTV > HRTV.

### 40C10 mAb protected against different genotypes of SFTSV infection in vivo

**Fig 4:**
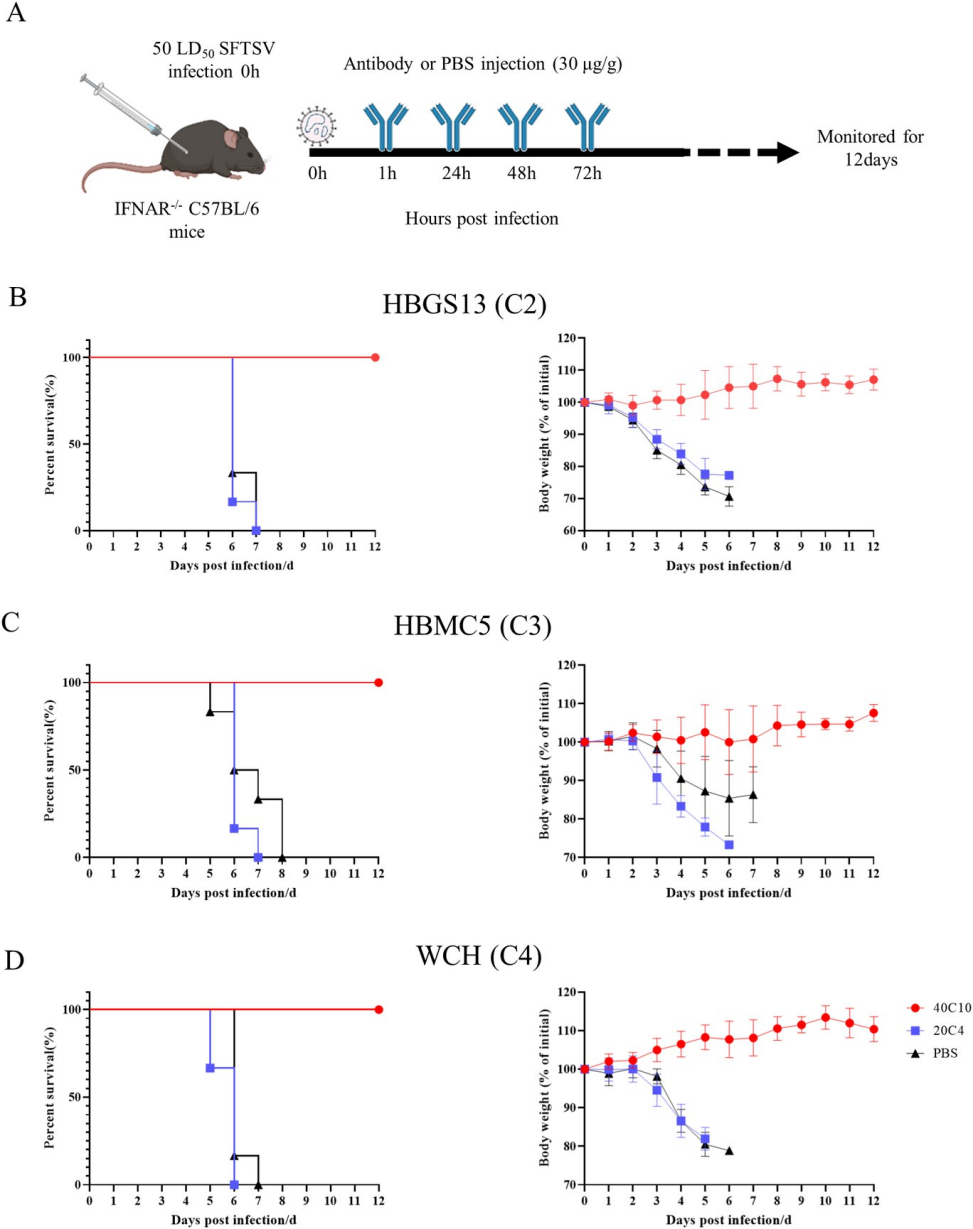
Administration of mAbs in IFNAR^-/-^ C57BL/6 mice infected with SFTSV of different genotypes. A. Overall scheme presenting the time points that mice were infected with SFTSV and administrated with mAbs. Mice were infected with SFTSV (50 LD_50_/mouse) and were given by 40C10, 20C4, or PBS mAb at 1, 24, 48 and 72 h p.i. at a dose of 30 μg/g. Survival rates (left) and body weight changes (right) were recorded in mice infected with SFTSV (B) HBGS13 (genotype C2), (C) HBMC5 (genotype C3), and (D) WCH (genotype C4) with treatment of 40C10, 20C4, or PBS. All data are presented as mean values ± SD.

When mice were infected with the HBGS13 (C2) strain, all mice in the 40C10 mAb group survived and their body weights ranged from 99.00% to 107.32%. In contrast, the control group injected with 20C4 mAb or PBS began to die on 6 d.p.i., and all mice died later. Moreover, the body weights of mice in the control group decreased significantly by 22.81% and 29.35%, respectively (Fig 4B). Similarly, when mice were infected with the HBMC5 (C3) or WCH (C4) strains, all mice in the 40C10 mAb group survived, with body weights ranging from 99.56% to 107.58% and 100% to 113.40%, respectively. In contrast, the control mice injected with the 20C4 mAb or PBS started to die on 5 d.p.i. and 6 d.p.i., respectively, resulting in the death of all mice. Additionally, the body weight of the mice in control group decreased significantly by 14.61% and 26.70% (HBMC5 strain), and by 18.06% and 21.12% (WCH strain), respectively (Fig 4C and 4D).

### 40C10 mAb reduced pathological changes in vivo

**Fig 5:**
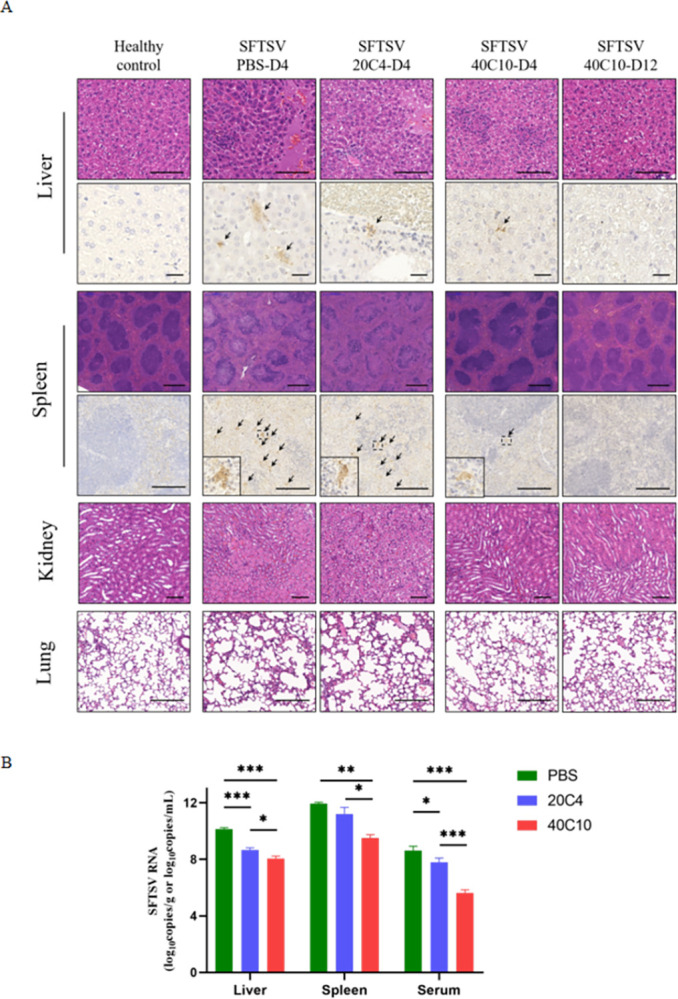
Evaluation of in vivo the protective abilities of the 40C10 mAb. HBGS13-C2 genotype of SFTSV was used to evaluate the protective abilities of the 40C10 mAb. The mice experiment was same as Fig 4A shows. Three mice from each group were dissected at 4 d.p.i. and three mice from group 40C10 mAb were dissected at 12 d.p.i., the blood, liver, spleen, lung and kidney were collected for evaluation. A. Pathological and immunohistochemical analysis of different tissue. The histopathologic changes of liver, spleen, lung and kidney were analyzed by hematoxylin-eosin staining. SFTSV antigen were detected by immunohistochemical experiment in liver and spleen tissue. Black arrow: positive for SFTSV NP antigen, different bar lengths in the Fig represent 100 μm. B. Viral RNA levels in liver, spleen and serum were measured by qRT-PCR assay at 4 d.p.i. (n  =  3 mice). All data are presented as mean values ± SD. Statistical differences were determined by t tests. *P < 0.05, **P < 0.01, ***P < 0.001.

At 4 d.p.i., the PBS and 20C4 mAb groups exhibited notable pathological features, including inflammatory infiltration, patchy liver bleeding, liver cell necrosis, atrophy or loss of white pulp in the spleen, renal interstitial bleeding, thickening of the alveolar septa, and enlarged lung vesicles (Fig 5). SFTSV NP antigens were detected in the liver and spleen. In contrast, the mice in 40C10 mAb group showed no apparent organ lesions at 4 d.p.i., with only mild inflammatory cell infiltration and a small amount of SFTSV NP antigen was detected in the liver. Furthermore, the mice in 40C10 mAb group recovered to normal levels at 12 d.p.i. (Fig 5). Meanwhile, at 4 d.p.i., the copies of viral RNA in serum, spleen, or liver in the 40C10 mAb group was significantly (p value 0.002–0.0313) lower than those in the PBS and 20C4 mAb group (Fig 5B). These findings suggest that the 40C10 mAb can effectively reduce organ damage and promote virus clearance both in blood and tissues.

### Delayed treatment with 40C10 mAb showed promising protection rates against lethal SFTSV infection

**Fig 6:**
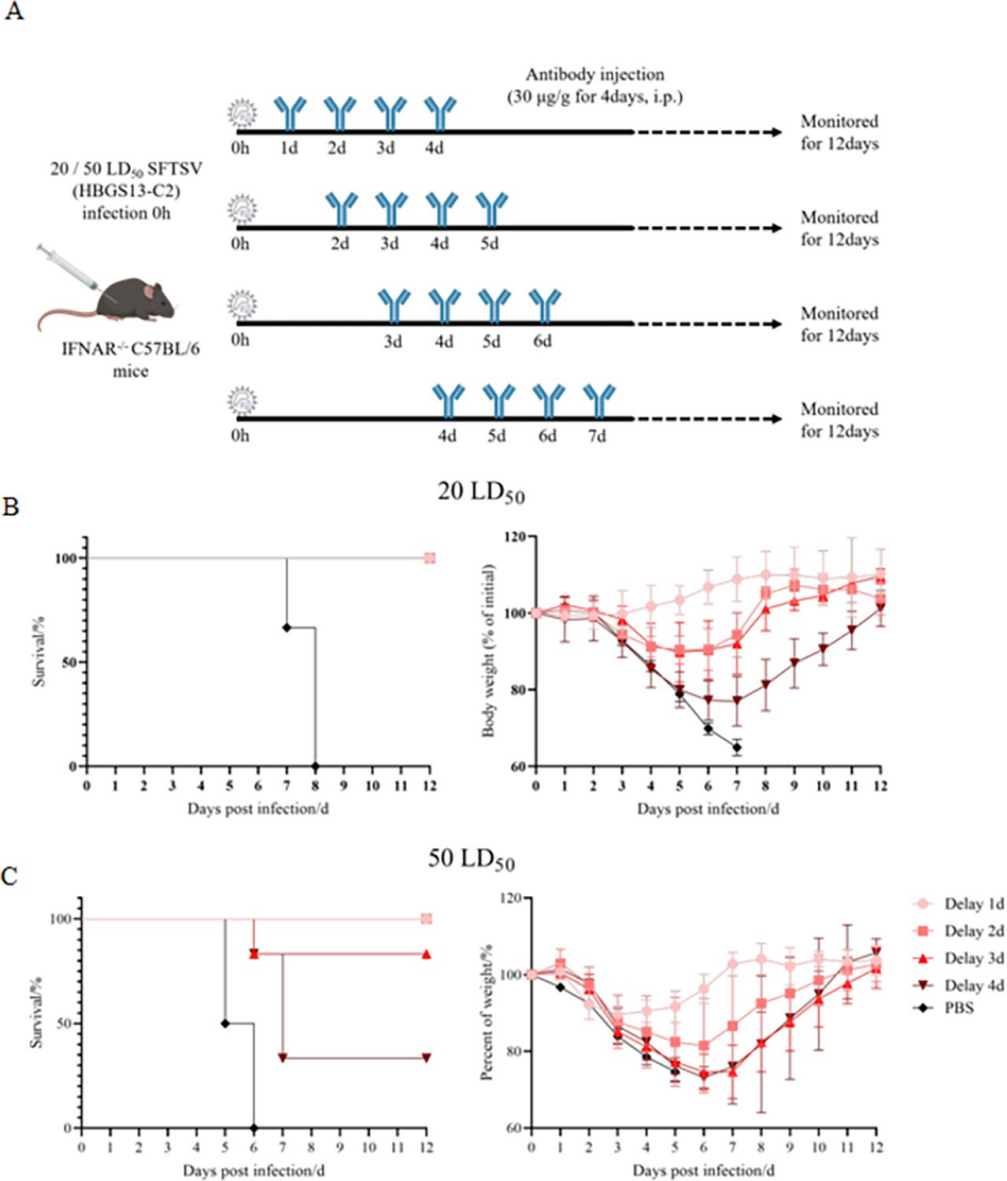
Evaluation of the protective effect of 40C10 given to mice infected with SFTSV using a delayed administration strategy. A. The diagram showing the strategy to give 40C10 to mice infected with SFTSV with the first infection after 1, 2, 3, or 4 days. The mice were infected with SFTSV (HBGS13, genotype C2) at a dose of 20 LD_50_ (B) or 50 LD_50_ (C), and their surviving rates (left) and body weight changes (right) were inspected daily for 12 days after infection. All data are presented as mean values ± SD.

Mice infected with a low dose (20 LD_50_) of SFTSV exhibited 100% survival, regardless of whether they received antibody treatment delayed for 1, 2, 3, or 4 days after infection (Fig 6B). The mice treated with antibodies since 1 d.p.i. showed minimal body weight loss, while the mice in other groups (2, 3, and 4-day delay) experienced weight lose of 10.19%, 10.19%, and 22.98%, respectively, but eventually regained their body weight, resulting in a 100% surviving rate. When a higher viral dose (50 LD_50_) was administered (Fig 6C), delaying 40C10 mAb treatment until 1 or 2 d.p.i. protected all mice, with a decrease in body weight of 11% or 19% respectively. A 3- or 4-day delay in 40C10 mAb administration protected 83.3% and 33.33% of mice respectively, indicating the potential of the 40C10 mAb to provide delayed protection. Notably, all mice injected with PBS at doses of 20 LD_50_ and 50 LD_50_ died.

## Discussion

Currently, there are vaccines or antivirals under development, but none are licensed [13,31]. Ribavirin and fapiravir can inhibit virus replication at the cellular and animal levels, and fapiravir demonstrated a benefit in terms of mortality only in patients with low viral loads on admission [32–34]. So far, two neutralizing mAbs against SFTSV have been identified deriving from SFTS patients and showed effective neutralization *in vitro* and *in vivo* [12,20]. Our previous study found that the differences in hotspots and sequences in the evolutionary performance of different genotype strains may affect the pathogenicity, infectivity, and immunogenicity of viruses [35]. Thus the development of broad-spectrum neutralizing mAbs is important for combating the widespread circulation of novel SFTSV strains and improving clinical treatment outcomes.

The SFTSV glycoprotein Gn/Gc facilitates viral entry by binding to cellular receptors [36]. Gn represents a target for neutralizing antibodies, indicating its significance in SFTSV prevention, vaccine design, and viral infection mechanism research [12]. In this study, we obtained three mAbs that recognize SFTSV-Gn, suggesting that Gn is an important antigen to induce antibody response. Previously literature has identified the protein epitope of SFTSV-Gn (E1-E5), which was recognized by SFTSV positive sheep serum [27]. The recognition of E2 and E3 epitopes by SFTS IgG-positive serum confirms their potential to induce antibody production, as previous reported [27].

Two neutralizing mAbs derived from patients with SFTS exhibited degrees of neutralization efficiency against different genotype SFTSV strains [12, 20]. MAb4-5, which targets the SFTSV-Gn domain III, demonstrated complete neutralization of C2 and C4 genotype strains at a concentration of 5 μg/mL [12]. On the other hand, Ab10 failed to neutralize the Gangwon/Korea/2012 strain (C2 genotype) even at a concentration of 50 μg/mL [20]. However, the software predictions indicated that Ab10 could recognize 247 out of the 272 analyzed strains, yielding an efficacy rate of 90.81% [20]. In this study, we found that 40C10 can effectively neutralize SFTSV Chinese clade strains (C1-C4 genotypes) *in vitro* (Fig 3), and it protected SFTSV (C2-C4 genotypes) infected-mice against disease and death (Fig 4). These findings indicated that the 40C10 has broad-spectrum antiviral effect. Given the limited number of reported C1 genotype SFTSV strains [35], we focused on evaluating the efficacy of 40C10 mAb in protecting mice from disease caused by C2-C4 genotype SFTSV strains.

Furthermore, 40C10 demonstrated neutralizing activity against other SFTSV-related viruses, namely HRTV and GTV, suggesting its potential as a candidate for clinical treatment of SFTSV-related virus infections. Unfortunately, due to the absence of suitable mouse models for HRTV and GTV infections, we were unable to assess the protective effects of the mAbs against these viruses. In the future, appropriate animal models for HRTV and GTV will be selected or established to evaluate the cross-protective effects of 40C10 mAbs. Additionally, we were unable to assess neutralization of the J clade due to the lack of Japanese clade strains. As 40C10 specifically targets the spatial structure of the Gn rather than linear epitopes, the antigenic sites of 40C10 were not determined in this study. Future investigations will employ structural biology techniques to analyze the spatial structure and interaction mechanisms between 40C10 and SFTSV-Gn. Furthermore, the human modification of the 40C10 antibody holds promise for advancing the development of broad-spectrum therapeutic neutralizing drugs and conducting antiviral research against SFTSV and related viruses.

In summary, we obtained three anti-SFTSV Gn mAbs based on hybridoma technology, and evaluated their ability to cross-recognize or cross-neutralize different strains of SFTSV and SFTSV-related viruses. We identified the epitopes recognized by the 18G4 and 20C4 mAbs, as well as their cross-reactivity with SFTSV-related viruses. The 40C10 mAb exhibited broad-spectrum neutralization activity against Chinese clade SFTSV strains, HRTV, and GTV, highlighting its efficacy against different bandaviruses. Furthermore, 40C10 showed signification cross-protection in mice infected with different genotypes SFTSV and displayed a delayed protective effect. Further investigation should focus on exploring the mechanism of action and potential application of this antibody through humanization modification and structural analyses.

## Supporting information

S1 Data Separate tables and separate word documents containing basic numerical data, statistical analysis and original pictures for Figs 1, 2B, 2C, 2D, 3A, 3B, 3E, 3F, 4B, 4C, 4D, 5A and 5B of this study. (RAR)
